# TROLLOPE: A novel sequence-based stacked approach for the accelerated discovery of linear T-cell epitopes of hepatitis C virus

**DOI:** 10.1371/journal.pone.0290538

**Published:** 2023-08-25

**Authors:** Phasit Charoenkwan, Sajee Waramit, Pramote Chumnanpuen, Nalini Schaduangrat, Watshara Shoombuatong

**Affiliations:** 1 Modern Management and Information Technology, College of Arts, Media and Technology, Chiang Mai University, Chiang Mai, Thailand; 2 Department of Zoology, Faculty of Science, Kasetsart University, Bangkok, Thailand; 3 Omics Center for Agriculture, Bioresources, Food, and Health, Kasetsart University (OmiKU), Bangkok, Thailand; 4 Center for Research Innovation and Biomedical Informatics, Faculty of Medical Technology, Mahidol University, Bangkok, Thailand; Emory University, UNITED STATES

## Abstract

Hepatitis C virus (HCV) infection is a concerning health issue that causes chronic liver diseases. Despite many successful therapeutic outcomes, no effective HCV vaccines are currently available. Focusing on T cell activity, the primary effector for HCV clearance, T cell epitopes of HCV (TCE-HCV) are considered promising elements to accelerate HCV vaccine efficacy. Thus, accurate and rapid identification of TCE-HCVs is recommended to obtain more efficient therapy for chronic HCV infection. In this study, a novel sequence-based stacked approach, termed TROLLOPE, is proposed to accurately identify TCE-HCVs from sequence information. Specifically, we employed 12 different sequence-based feature descriptors from heterogeneous perspectives, such as physicochemical properties, composition-transition-distribution information and composition information. These descriptors were used in cooperation with 12 popular machine learning (ML) algorithms to create 144 base-classifiers. To maximize the utility of these base-classifiers, we used a feature selection strategy to determine a collection of potential base-classifiers and integrated them to develop the meta-classifier. Comprehensive experiments based on both cross-validation and independent tests demonstrated the superior predictive performance of TROLLOPE compared with conventional ML classifiers, with cross-validation and independent test accuracies of 0.745 and 0.747, respectively. Finally, a user-friendly online web server of TROLLOPE (http://pmlabqsar.pythonanywhere.com/TROLLOPE) has been developed to serve research efforts in the large-scale identification of potential TCE-HCVs for follow-up experimental verification.

## 1. Introduction

Hepatitis C Virus (HCV) is an RNA virus that is associated with progressive liver damage. This virus usually transmits through the contact of blood from an infected person, including the reuse of substandard medical equipment in healthcare settings and the sharing of contaminated needles and syringes in injection drug users (IDU). HCV infection often leads to curable acute hepatitis C; however, it can also result in an asymptomatic chronic condition that can lead to serious illnesses, including liver fibrosis, cirrhosis, and even fatal hepatocellular carcinoma [1, 2]. By combining translational and clinical research efforts, treatments for HCV have evolved from recombinant interferon α (IFNα) and nucleoside analogue ribavirin (RBV) to direct-acting antiviral agents (DAAs). These treatment options can be administered as monotherapy or in combination to achieve superior outcomes [3, 4]. Currently, treatment approaches primarily focus on pan-genotypic regimens designed to target multiple viral non-structural (NS) complexes that aim to achieve high efficiency in treating most HCV genotypes [5, 6]. Despite the success of HCV therapeutic schemes, access to diagnosis and treatment remains limited in certain populations. According to the World Health Organization (WHO), approximately 58 million people worldwide are affected by chronic HCV infection, yet only 21% of them have been clinically diagnosed. Additionally, the disease is also responsible for approximately 400,000 deaths each year due to cirrhosis and hepatocellular carcinoma [7, 8]. This is considered inconsistent with the announcement to reduce new HCV infections by 90% by 2030 and achieve complete HCV elimination as the ultimate goal. According to WHO, the number of diagnosed HCV patients is underestimated, and access to the tests is still limited in some populations. Therefore, HCV vaccine development is essentially required to prevent transmission. An effective HCV vaccine will greatly impact the control of the disease, especially among IDUs. It is worth noting that the process of vaccine production should be cost-effective to ensure worldwide fair access. Despite substantial positive outcomes in treating HCV patients, the lack of available preventative vaccines hinders significant progress toward the goal of HCV elimination [9].

One major challenge in vaccine development is the genetic diversity of HCV, which consists of 8 genotypes and 86 subtypes. These variants exhibit approximately 30 percent variability in amino acids compared to each other [10]. To achieve the greatest benefit, an ideal vaccine should focus on targeting the genetically conserved regions of the HCV genome. This approach would broaden the immune response across multiple genotypes and involve both humoral and cellular immunity, thereby maximizing the chance of success. Many studies have demonstrated the feasibility of neutralizing HCV infection through the transfer of polyclonal antibodies obtained from chronic HCV patients to chimeric mice and chimpanzees [11–13]. Nonetheless, the envelope genes (E1 and E2) of HCV exhibit significant diversity, resulting in a wide range of evolved epitopes that are resistant to antibody binding. This evolutionary adaptation benefits the viral escape from immune responses [14–16]. Additionally, the specific roles of antibodies in combating HCV infection have not been clearly defined yet, which further complicates the development of antibody-based vaccines [16]. In contrast, several vaccine studies have concentrated on enhancing HCV-specific T-cell activity [17]. Specifically, CD4^+^ T cells play a crucial role in maintaining T-cell populations, while CD8^+^ T cells serve as the primary effectors responsible for eliminating viral-infected cells [18–21]. To date, various non-structural proteins (NS) of HCV have been found to possess prominent targeting features for CD8^+^ T cells, indicating the feasibility of vaccine development. However, this strategy essentially relies on the presence of HCV antigenic peptides in an HLA-restricted manner [22, 23].

Until now, several HCV vaccine platforms, such as DNA-based immunization, virus-like particles (VLPs), and short peptide- or epitope-encapsulating lysosomes [24–26], have demonstrated promising outcomes in terms of HCV protection. However, some of these still need particular improvements to enhance their effectiveness [27, 28]. One of the challenges lies in the rational design of immunogenic epitopes [29], as the traditional vaccine design approaches are considered less effective for HCV due to high genome heterogeneity and mutagenicity [30]. Thus, an alternative approach is required, and *in silico* studies have shown great benefits by predicting immunogenic epitopes to be incorporated into the vaccine platform and enhance its efficacy. Many *in silico* predictions of TCE-HCV have demonstrated promising outcomes in terms of cytotoxic T cell responses in BALB/c and transgenic mice. These findings suggest the potential of HCV-polytope vaccine candidates. However, some of the predicted epitopes have achieved only marginal success and require additional support [31–33]. Therefore, it is desirable to accurately identify TCE-HCV using sequence information alone, without relying on structural information, before embarking on costly *in vitro* and *in vivo* investigations.

To date, several computational approaches have been developed to complement experimental studies in the identification of TCEs. For example, Dhanda et al. [34] developed a support vector machine (SVM)-based predictor, named IL4pred, to predict IL4 inducing peptides. They constructed a benchmark dataset consisting of 904 IL4 inducing and 742 non-IL4 inducing peptides. Using this dataset, various sequence-based feature descriptors were employed, such as amino acid composition (AAC), amino acids pair (AAP), dipeptide composition (DPC), and motif information, to train IL4pred. Among the feature descriptors, AAP and motif information were selected for the development of IL4pred. IL4pred achieved cross-validation and independent test accuracies of 0.758 and 0.690, respectively. Further information regarding related computational approaches developed for the identification of TCEs can be found in references [35–37]. However, at present, there is no sequence-based predictor specifically designed for identifying and characterizing TCE-HCVs. Keeping this issue in mind, we present a novel sequence-based stacked approach, termed TROLLOPE (predicToR Of Linear t-ceLl epitOPEs of hepatitis C virus), to specifically identify TCE-HCVs using primary sequence information. To the best of our knowledge, TROLLOPE is the first computational approach developed for specifically identifying TCE-HCVs. To develop TROLLOPE, we first constructed a benchmark dataset consisting of 446 TCE-HCVs and 525 non-TCE-HCVs. Based on this dataset, we extracted 12 different types of sequence-based feature encoding schemes from several perspectives, such as physicochemical properties, composition-transition-distribution information and composition information. These feature descriptors were then used to create 144 base-classifiers by using 12 powerful ML algorithms. To maximize the performance of TROLLOPE, we employed a customized genetic algorithm to determine a collection of potential base-classifiers and integrated them to develop the meta-classifier using the stacking strategy. Experimental results demonstrate that TROLLOPE outperforms conventional ML classifiers, achieving superior performance.

## 2. Materials and methods

### 2.1 Overall framework of TROLLOPE

**Fig 1:**
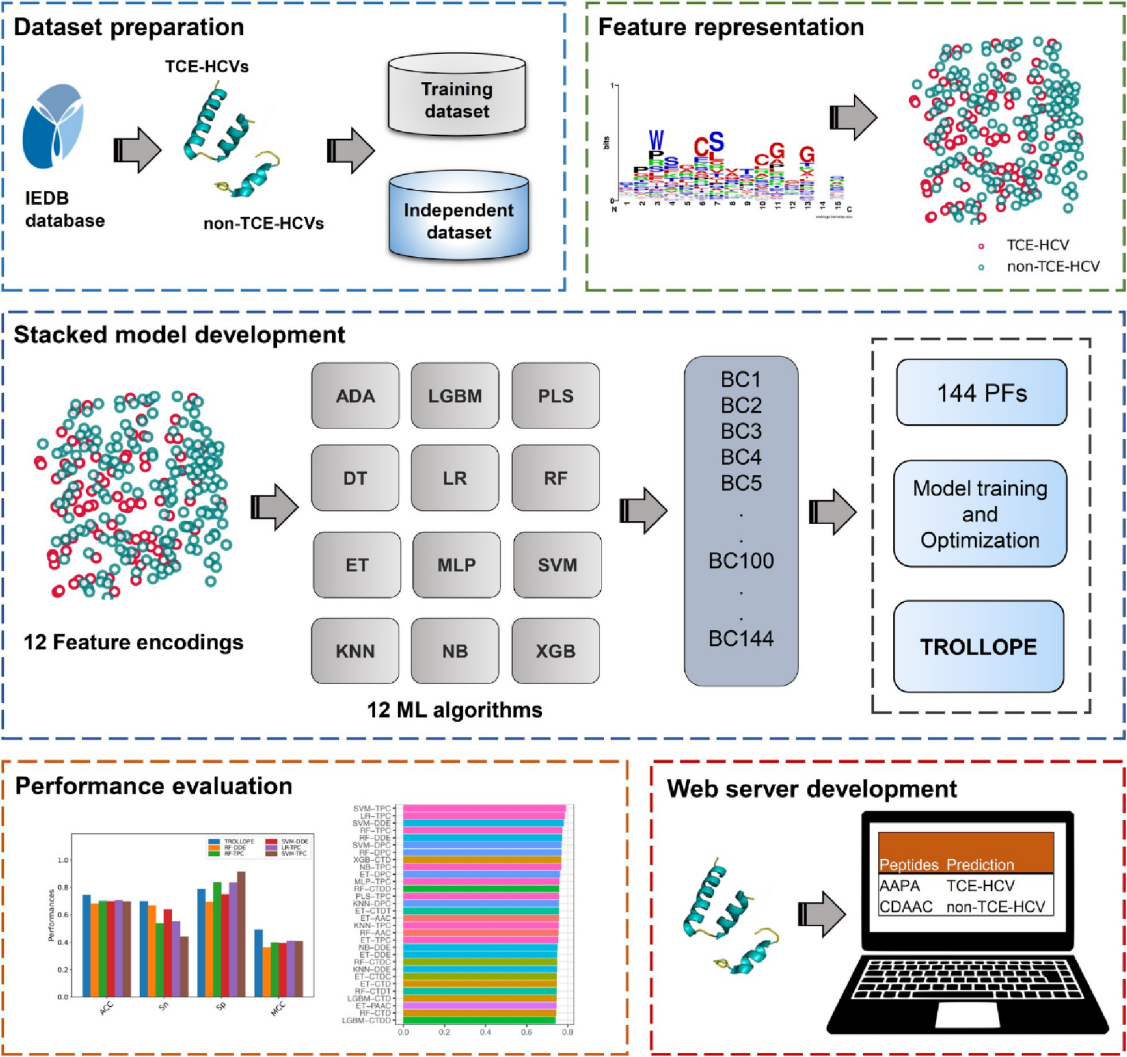
System flowchart of the proposed TROLLOPE. The development and performance assessment of TROLLOPE involves five main steps: dataset preparation, feature representation, stacked model development, performance evaluation, and online web server deployment.

As seen in **Fig 1**, the development and performance assessment of TROLLOPE involve five main steps: dataset preparation, feature representation, stacked model development, performance evaluation, and online web server deployment. In the first step, we collected the positive and negative datasets from the IEDB database [38]. In the second step, we employed well-known feature encoding schemes to represent TCE-HCVs and non-TCE-HCVs. After that, in the development of stacked model, it consists of two levels of learning stages. The classifiers developed in the first and second stages are known as the base-classifier and meta-classifier, respectively. In the fourth step, we assessed the performance of base-classifiers and meta-classifiers to conduct a comparative analysis and select the final stacked model. Finally, the best stacked model was employed to develop an online web server, providing convenient identification of TCE-HCVs.

### 2.2 Data collection and curation

According to the previously established B-cell response of the HCV dataset [39], the positive and negative samples were collected from Immune Epitope Database (IEDB) version 2.26. Specifically, the dataset used in this study was created by selecting “Hepatitis C virus” (ID 11103) as the source organism. The main criteria for the inclusion of experimental outcomes were related to T cell assays in human, mouse and non-human primates. Among these, peptide sequences annotated as ‘positive’ were considered as the positive samples (referred to as TCE-HCVs) whereas those annotated as ‘negative’ were included as the negative samples (referred to as non-TCE-HCVs). The length of peptide sequences was filtered to 8–10 amino acid residues to select compatible epitopes capable of being accommodated through HLA I molecules and recognizing by CD8^+^ T cells [40, 41]. As a result, we obtained 711 TCE-HCVs and 790 non-TCE-HCVs. Among these sequences, redundant samples were excluded using Venny (https://bioinfogp.cnb.csic.es/tools/venny/index.html). Therefore, 446 TCE-HCVs and 525 non-TCE-HCVs were considered as the benchmark dataset and used for constructing the proposed model. Finally, the benchmark dataset was randomly divided to generate the training and independent test datasets, comprising 80% (i.e., 357 TCE-HCVs and 420 non-TCE-HCVs) and 20% (i.e., 89 TCE-HCVs and 105 non-TCE-HCVs) of the data, respectively.

### 2.3 Informative feature selection

In this study, our customized genetic algorithm, called GA-SAR [42], was employed to determine informative features while securing high prediction performance [42–45]. In general, GA-SAR is categorized as one of commonly-used non-deterministic methods that utilize the biological evolution of a population [46, 47]. The GA-SAR’s chromosome used herein comprises binary for feature selection and parametric genes for ML parameter optimization. The feature importance selection based on the GA-SAR method can be described as follows. First, we randomly constructed an initial population of *Pop* individuals and assess the performance of all *Pop* individuals based on the 10-fold cross-validation test. Second, we utilize a commonly-used tournament selection to obtain the best *Pop* for constructing a mating pool. Third, the self-assessment-report operation (SAR) between the best *Pop* and each other individual *Pop* was used to create the new children. Finally, the maximum number of generations is used as the stopping condition. Herein, the parameters and their values for the GA-SAR contain *r*_*begin*_  =  5, *m*_*stop*_   =   20, *P*_*m*_  =   0.05, and *Pop* = 20 [44, 48, 49]. Detailed information about this algorithm is reported in our previous studies [42, 44, 48].

### 2.4 Stacked model development

Ensemble learning strategies integrate heterogeneous outputs from different prediction models to create a single prediction. These strategies include average scoring, majority voting, and the stacking strategy [50, 51]. Among these strategies, stacking is known as the most powerful one [49–53]. This approach was first presented by Wolpert [54] to improve prediction performance. Therefore, we employed the stacking strategy to develop TROLLOPE. In general, the stacking ensemble framework consists of two main levels of learning stages, where the prediction models developed from the first and second learning stages are referred to as the base-classifier and meta-classifier, respectively. The design and development process of TROLLOPE is illustrated in **Fig 1**.

In the first learning stage, we employ 12 different ML algorithms (ADA, DT, ET, KNN, LGBM, LR, MLP, NB, PLS, RF, SVM, and XGB) to obtain the crucial pattern of TCE-HCV [51, 55, 56]. Then, each ML algorithm was train with 12 well-known feature descriptors (AAC, AAI, APAAC, CTD, CTDC, CTDD, CTDT, DDE, DPC, PAAC, PCP and TPC [57–60]) to construct 12 base-classifiers. The details of all the feature encodings and ML methods used herein are recorded in **Table 1** and **S1 Table in S1 File**, respectively. As a result, 144 base-classifiers were obtained by using the Scikit-learn package in Python programming language [61]. Specifically, a grid search based on the 10-fold cross-validation procedure was used to determine the optimal parameters of all the 144 base-classifiers and avoid overfitting. Here, the base-classifiers having the highest area under the receiver operating characteristics (ROC) curve (AUC) were deemed as the powerful classifiers.

**Table 1:** Summary of 12 different feature descriptors along with their corresponding description and dimension.

Descriptors^a^	Description	Dimension	Reference
AAC	Frequency of 20 amino acids	20	[90]
AAI	All biochemical and biophysical properties extracted from the AAindex database	531	[48]
APAAC	Amphiphilic pseudo-amino acid composition	22	[91, 92]
CTD	Composition, transition and distribution	273	[90]
CTDC	Percentage of particular amino acid property groups	21	[90, 93, 94]
CTDD	Percentage of mutual conversion in amino acid properties	21	[90, 93, 94]
CTDT	Distribution of amino acid properties in sequences	105	[90, 93, 94]
DDE	Dipeptide deviation from expected mean	400	[95]
DPC	Frequency of 400 dipeptides	400	[95]
PAAC	Pseudo amino acid composition	21	[91, 92]
PCP	Selected important physical and chemical properties	11	[48]
TPC	Frequency of 8000 tripeptides	8000	[50, 95]

^a^AAC: amino acid composition, AAI: amino acid composition and physicochemical properties, APAAC: pseudo amino acid composition, CTD: composition translation and distribution, CTDC: CTD composition, CTDT: CTD distribution (CTDT), CTDT: CTD transition (CTDT), DDE: dipeptide deviation from expected mean, DPC: dipeptide composition, PAAC: pseudo amino acid composition, PCP: physicochemical properties, TPC: tripeptide composition.

In the second step, we utilized each base-classifier to generate a probabilistic feature (PF) exhibiting the probabilistic score of being TCE-HCV. To be specific, we randomly divided the training dataset into 10 subsets (i.e., $D_{TRN}=\{D_{1},D_{2},D_{3},\ldots ,D_{i}\},where\;i=1,2,3,\ldots ,10$) based on the 10-fold cross-validation procedure. In the stacking strategy, each D_i_ was treated as the validation set, while the remaining nine subsets was treated as the training set, which was used for training a subset-based prediction model. Then, 10 subset-based prediction models were obtained and used to calculate 10 different probabilistic scores for each peptide sequence on the independent test dataset. Thus, the 10 different probabilistic scores were averaged to create the PF. As a result, 144 PFs derived from all the 144 base-classifiers were obtained and used to construct a new probabilistic feature vector (referred to as APF). For a given sequence *P*, its probabilistic feature vector can be represented as follows:

$$APF(P)=\{{PF}_{1,1},{PF}_{1,2},{PF}_{1,3},\ldots ,{PF}_{i,j},\ldots ,{PF}_{i,j}\},where\;i,j=1,2,3,\ldots ,12$$ (1)

where PF_i,j_ is the probabilistic feature generated by the base-classifier trained with the *i*^*th*^ ML algorithm in conjunction with the *j*^*th*^ feature encoding. Finally, the APF is represented with a 144-D probabilistic feature vector.

In the third learning stage, we utilized the APF to train the PLS-based meta-classifier. In the meanwhile, to enhance the performance of the meta-classifier, we used the GA-SAR method to determine *m* informative PFs, where *m* << 144. The GA-SAR’s chromosome used herein comprises 144 genes and 10-bit gene for encoding #Components of PLS-based meta-classifier, where #Components ∈ {10, 20, 30, 40, …, 1000} (**S1 Table in S1 File**). By doing this, we obtained a *m*-D probabilistic feature vector (referred to as OPF) generated from the selected base-classifiers. Finally, we obtained two PLS-based meta-classifiers and selected the best-performing one for TROLLOPE construction based on the cross-validation AUC.

### 2.5 Statistical metrics

The performance of the proposed model and related conventional ML classifiers was determined using five standard evaluation metrics, including AUC, sensitivity (Sn), specificity (Sp), accuracy (ACC), and Matthew’s correlation coefficient (MCC) [62–64]. These evaluation metrics are computed as follows:

$$Sn=\frac{TP}{\left(TP+FN\right)}$$ (2)

$$Sp=\frac{TN}{\left(TN+FP\right)}$$ (3)

$$ACC=\frac{TP+TN}{\left(TP+TN+FP+FN\right)}$$ (4)

$$MCC=\frac{TP\times TN-FP\times FN}{\sqrt{\left(TP+FP)(TP+FN)(TN+FP)(TN+FN\right)}}$$ (5)

where TN represents true negatives (e.g., the number of correctly predicted non-TCE-HCV) and TP represents true positives (e.g., the number of correctly predicted TCE-HCVs). On the other hand, FN represents false negatives (e.g., the number of TCE-HCVs predicted as non-TCE-HCVs), while FP represents false positives (e.g., the number of non-TCE-HCVs predicted as TCE-HCVs).

## 3. Results and discussion

### 3.1 Performance evaluation of different feature encodings and ML methods

**Fig 2:**
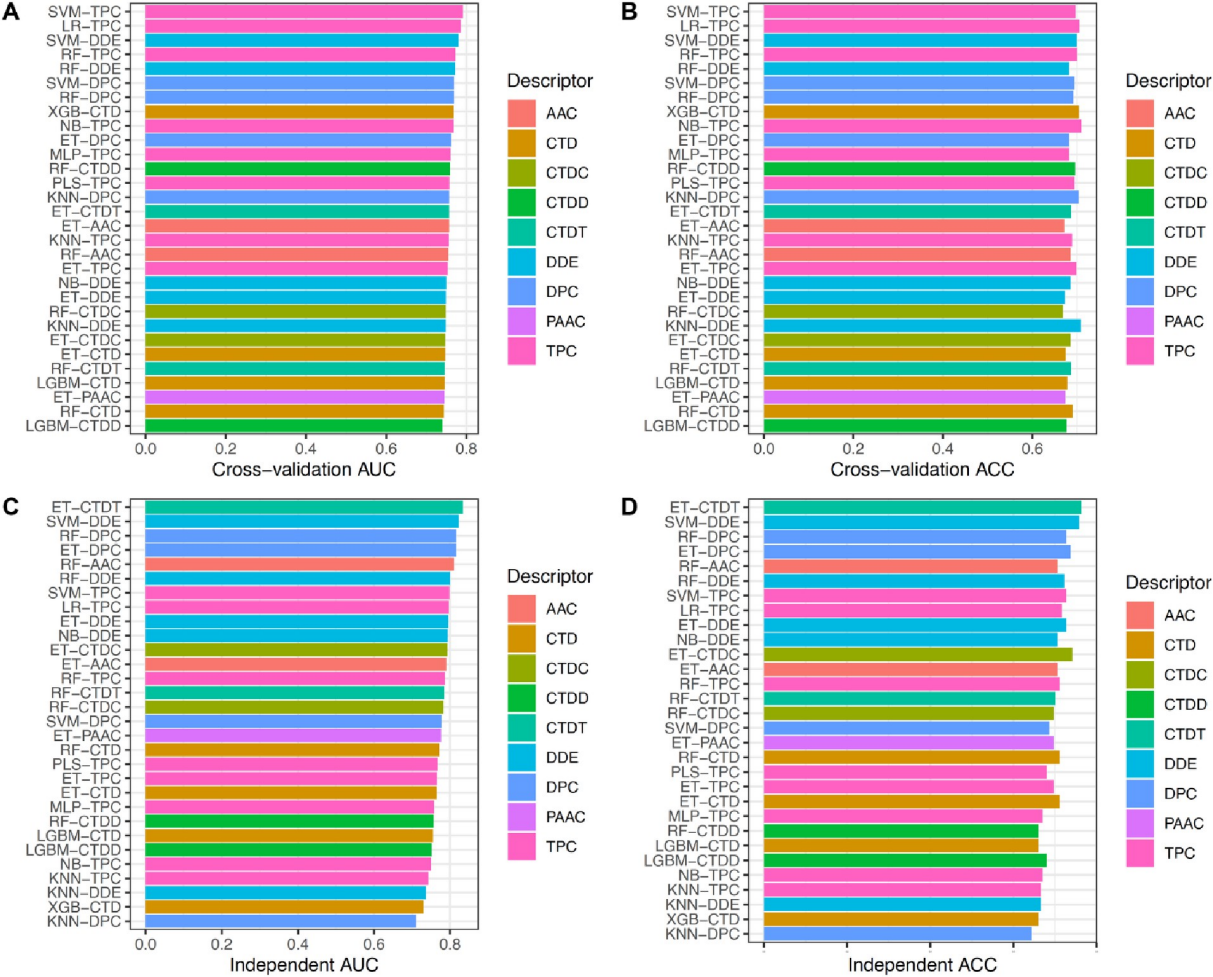
Performance evaluations of top-30 base-classifiers. (**A-B**) Cross-validation AUC and ACC of top-30 base-classifiers. (**C-D**) Independent AUC and ACC of top-30 base-classifiers.

**Fig 3:**
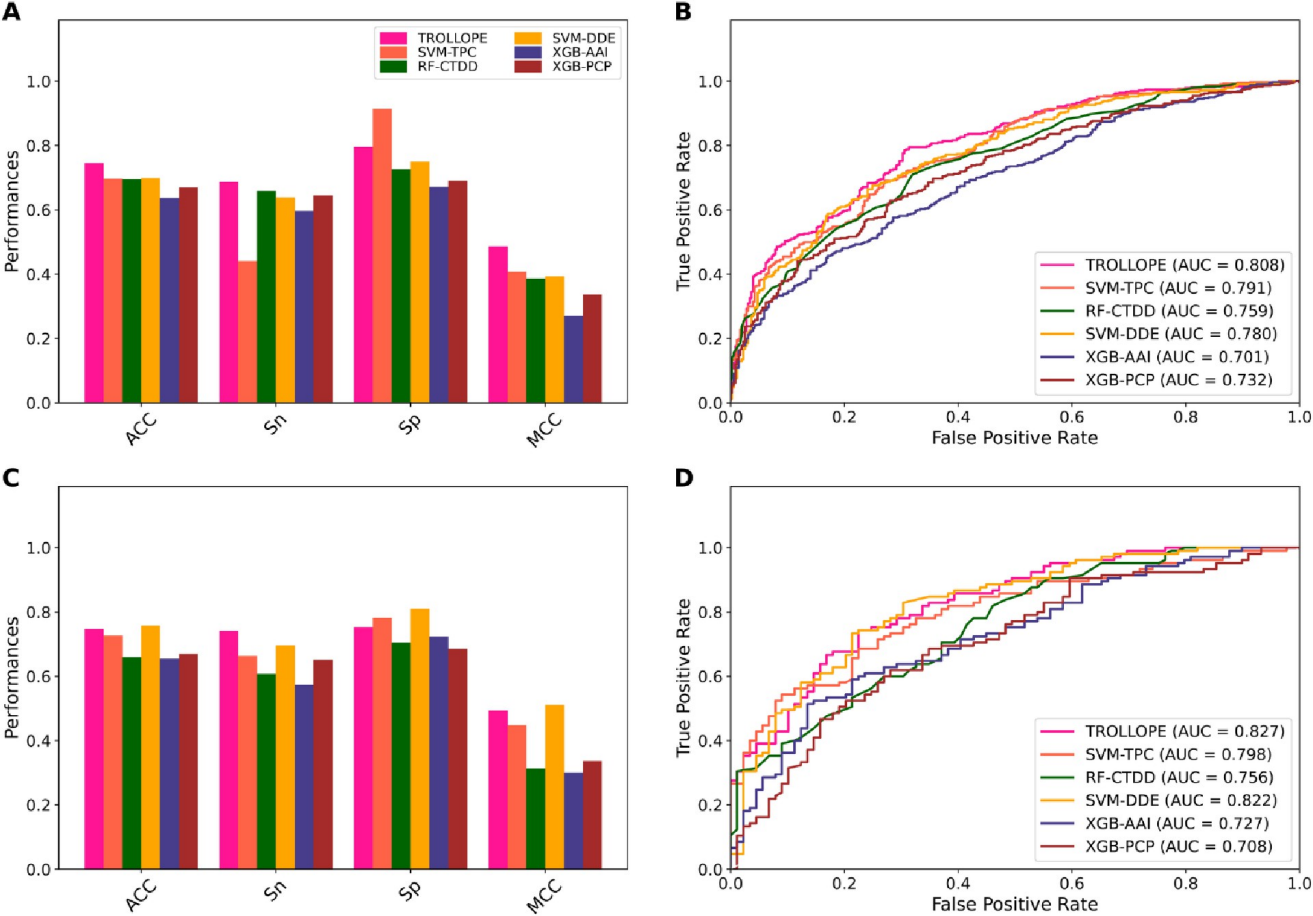
Performance comparison of TROLLOPE and top-five base-classifiers on the training (**A–B**) and independent (**C–D**) datasets.

In this section, we investigated the prediction capability of various base-classifiers trained with different feature encodings and ML methods in TCE-HCV prediction. For each base-classifier, we evaluated its performance using both the 10-fold cross-validation and independent tests. As mentioned earlier, we determined the best-performing base-classifiers in terms of cross-validation AUC. The detailed results of the10-fold cross-validation and independent tests for all the 144 BCs are recorded in **Figs 2 and 3** and **S2, S3 Tables in S1 File**. From **Fig 2**, we notice that 8 out of the 10 top-ranked powerful base-classifiers were developed based on DDE, DPC, and TPC, i.e., SVM-TPC, LR-TPC, SVM-DDE, RF-TPC, RF-DDE, SVM-DPC, RF-DPC, NB-TPC, and ET-DPC with corresponding AUC values of 0.791, 0.786, 0.780, 0.772, 0.771, 0.769, 0.769, 0.768, 0.762, respectively. This indicates that these three feature descriptors are beneficial in TCE-HCV prediction. Interestingly, the AUC values of SVM-TPC and LR-TPC were over 0.780 in terms of the 10-fold cross-validation test. It could be stated that SVM-TPC is deemed as the best-performing classifier in TCE-HCV prediction. As seen in **S2 Table in S1 File**, the ACC, Sn, Sp, and MCC of SVM-TPC were 0.696, 0.440, 0.914, and 0.407, respectively. On the other hand, this base-classifier achieved the eighth highest AUC of 0.798 in the independent test results, while the highest AUC of 0.833 was achieved by ET-CTDT (**S3 Table in S1 File**). These results demonstrate that the single feature-based models provide a less stable performance for TCE-HCV prediction. Thus, we were motived to develop a stacked model by integrating heterogonous ML classifiers in order to yield a more accurate and stable TCE-HCV prediction.

### 3.2 Construction and optimization of stacked models

As mentioned above, we developed RF-based meta-classifiers that cooperate with two new probabilistic feature vectors, namely APF and OPF. The APF and OPF are represented with 144-D and *m*-D probabilistic feature vectors, respectively. The optimal number of *m* out of 144 probabilistic features was determined using the GA-SAR method. After optimizing the 144-D probabilistic feature vector, the optimal number of *m* was found to be 6. Specifically, the top-six informative probabilistic features were generated based on six different base-classifiers, inducing SVM-TPC, RF-CTDD, SVM-DDE, XGB-AAI, XGB-PCP, and ET-APAAC. The performance of the APF and OPF was evaluated based on both the 10-fold cross-validation and independent tests (**Table 2**). As can be seen from **Table 2**, the OPF outperforms APF in terms of the 10-fold cross-validation results, with a 4.90% increase in ACC, a 4.21% increase in Sn, a 5.48% increase in Sp, a 9.75% increase in MCC, and a 1.65% increase in AUC. In terms of the performance on the independent test dataset, the OPF achieved the best AUC, ACC, and MCC with an increase of 1.11, 1.03 and 2.42%, respectively. Altogether, the OPF in conjunction with the RF-based meta-classifier was selected for the construction of TROLLOPE.

**Table 2:** Cross-validation and independent test results of stacked models trained with APF and OPF feature vectors.

Evaluation strategy	Feature	Dimension	ACC	Sn	SP	MCC	AUC
Cross-validation	APF	144	0.696	0.644	0.740	0.389	0.792
	OPF	15	0.745	0.686	0.795	0.487	0.808
Independent test	APF	144	0.737	0.652	0.810	0.469	0.816
	OPF	15	0.747	0.742	0.752	0.493	0.827

### 3.3 Performance comparison between TROLLOPE and related ML methods

**Fig 4:**
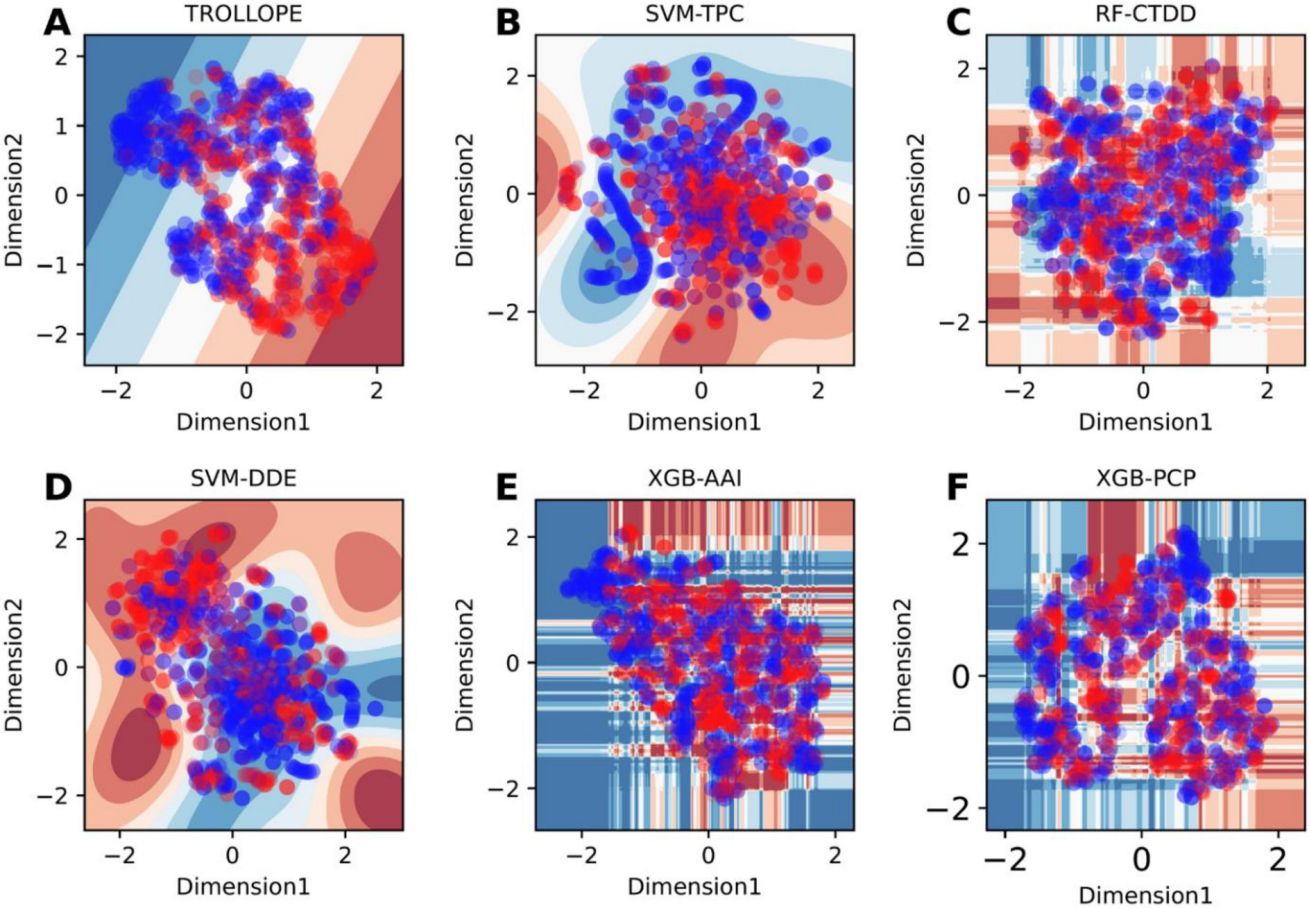
t-distributed stochastic neighbor embedding (t-SNE) distribution of positive and negative samples on the training dataset, where TCE-HCV and non-TCE-HCV are represented with red and blue dots, respectively. TROLLOPE (**A**) and top-five base-classifiers (**B-F**).

To reveal the effectiveness of our proposed model TROLLOPE, we compared its performance with related ML methods. However, there is no existing computational model designed for TCE-HCV identification. Thus, the performance of TROLLOPE is compared with related ML methods, involving BLAST-based predictor, two well-known ensemble strategies (i.e., the average scoring and majority voting), and its base-classifiers, in terms of both 10-fold cross-validation and independent tests. Firstly, we compared the performance of TROLLOPE with the BLAST-based predictor. The BLAST-based predictor is a well-known similarity-based approach for identifying proteins [65]. **S4 Table in S1 File** summarizes the independent test results of the BLAST-based predictor based on different E-values. As can be seen from **S4 Table in S1 File**, TROLLOPE clearly outperforms the BLAST-based predictor in terms of ACC, Sn, Sp, and MCC. Secondly, we conducted a comparative experiment between TROLLOPE and the selected ensemble strategies. **Table 3** provides the comparative results of the three ensemble strategies. We noticed that both cross-validation and independent test results of TROLLOPE were better than that of the two compared ensemble strategies in terms of all five measures, with the exception of Sp on the independent test dataset. To be specific, the AUC of TROLLOPE were 2.93–2.97% and 2.56–3.33% better than that of the two related ensemble strategies in terms of cross-validation and independent tests, respectively, highlighting the effectiveness of the stacking strategy over other ensemble strategies. Finally, the performance of TROLLOPE was compared against its constituent base-classifiers. For convenience of discussion, we selected the top-five base-classifiers (i.e., SVM-TPC, LR-TPC, SVM-DDE, RF-TPC, and RF-DDE) for conducting our comparative results. From **Figs 3 and 4** and **Table 4** along with **S1 Fig in S1 File**, several observations can be summarized as follows: (i) TROLLOPE attains the overall best cross-validation results in terms of ACC, Sn, MCC, and AUC; (ii) The ACC, MCC, and AUC of TROLLOPE are higher than most top-five base-classifiers in terms of the independent test dataset, with the exception of SVM-DDE; (iii) TROLLOPE demonstrates a significant improvement, achieving a 2.06% increase in ACC, a 7.87% increase in Sn, a 4.52% increase in MCC, and a 2.92% increase in AUC compared to the best-performing base-classifier (i.e., SVM-TPC); and (iv) Based on the distributed stochastic neighbor embedding (t-SNE) method [66, 67], TROLLOPE demonstrates greater discriminative power in making accurate predictions (**Fig 4**). Overall, our comparative analysis revealed that the stacking strategy used in TROLLOPE proved beneficial in terms of providing more accurate and reliable identification of TCE-HCV.

**Table 3:** Performance comparison of different models trained based on different ensemble strategies.

Evaluation strategy	Ensemble strategy	ACC	Sn	Sp	MCC	AUC
Cross-validation	Average score	0.690	0.647	0.726	0.374	0.779
	Majority voting	0.689	0.613	0.752	0.370	0.779
	Stacking	0.745	0.686	0.795	0.487	0.808
Independent test	Average score	0.727	0.674	0.771	0.448	0.801
	Majority voting	0.706	0.607	0.790	0.405	0.794
	Stacking	0.747	0.742	0.752	0.493	0.827

**Table 4:** Performance comparison of TROLLOPE and top-five ML classifiers.

Evaluation strategy	Method	ACC	Sn	Sp	MCC	AUC
Cross-validation	RF-DDE	0.682	0.669	0.693	0.363	0.771
	RF-TPC	0.700	0.538	0.838	0.399	0.772
	SVM-DDE	0.699	0.639	0.750	0.394	0.780
	LR-TPC	0.705	0.552	0.836	0.409	0.786
	SVM-TPC	0.696	0.440	0.914	0.407	0.791
	TROLLOPE	0.745	0.686	0.795	0.487	0.808
Independent test	RF-DDE	0.722	0.753	0.695	0.447	0.799
	RF-TPC	0.711	0.517	0.876	0.426	0.786
	SVM-DDE	0.758	0.697	0.810	0.511	0.822
	LR-TPC	0.716	0.539	0.867	0.434	0.796
	SVM-TPC	0.727	0.663	0.781	0.448	0.798
	TROLLOPE	0.747	0.742	0.752	0.493	0.827

### 3.4 Analysis of the contribution of new probabilistic features

**Fig 5:**
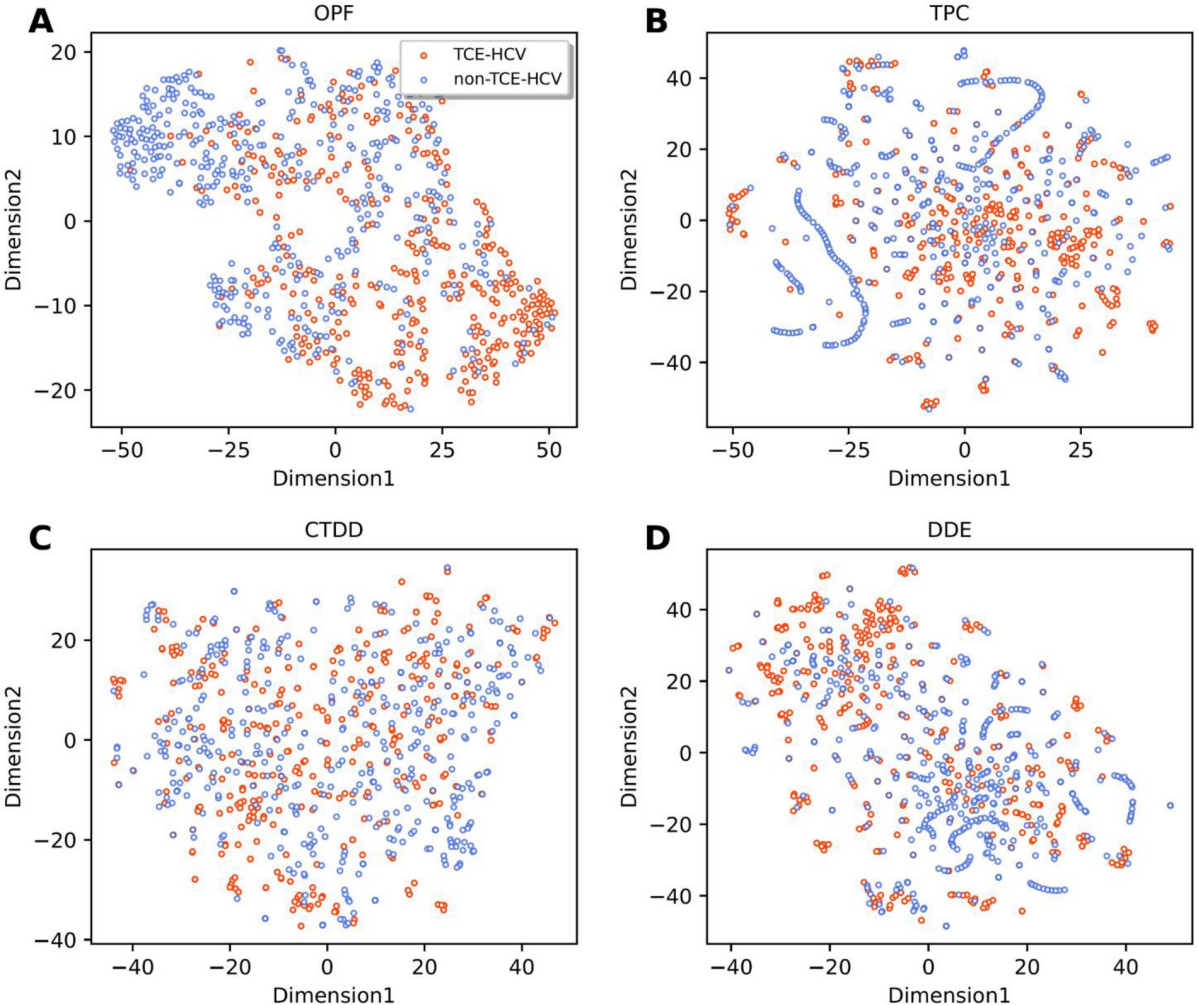
t-SNE plots of our new feature OPF (**A**) and top-three feature descriptors (**B-D**) (i.e. DDE, DPC, and TPC) on the training dataset.

In this section, we investigated the discriminative power of the new probabilistic features (OPF) for identifying TCE-HCVs. Firstly, we compared the performance of OPF with well-known sequence-based feature descriptors, involving AAC, AAI, APAAC, CTD, CTDC, CTDD, CTDT, DDE, DPC, PAAC, PCP and TPC. By doing this, each feature was used to develop a PLS-based model and its performance was evaluated based on the 10-fold cross-validation and independent tests. **Tables 5 and 6** record their detailed 10-fold cross-validation and independent test results. As seen from **Table 5**, among the 12 well-known feature descriptors, the highest AUC of 0.772 is achieved using TPC. This indicates that TPC exhibits greater discriminative power compared to other feature descriptors. Furthermore, **Tables 5 and 6** show that the OPF achieves higher ACC, Sn, MCC, and AUC values compared to 12 well-known feature descriptors in terms of both the 10-fold cross-validation and independent tests. Impressively, on the independent test dataset, the OPF outperforms TPC in terms of MCC, Sn, ACC, and AUC, with an increase of 13.84, 11.24, 6.70, and 6.00%, respectively. Secondly, we employed the t-SNE method to analyze the feature space of OPF and top-three informative sequence-based feature descriptors (i.e., DDE, DPC, and TPC) to visualize their distributions. As can be seen from **Fig 5**, the feature space derived from OPF exhibits clearer and more distinct clusters as compared to DDE, DPC, and TPC. Our comparative analysis revealed that our new probabilistic features exhibited more discriminative power in identifying TCE-HCV compared to well-known sequence-based feature descriptors, resulting in improved performance.

**Table 5:** Cross-validation results of our new feature and conventional feature descriptor.

Feature	ACC	Sn	Sp	MCC	AUC
CTDC	0.538	0.401	0.655	0.056	0.558
PCP	0.538	0.325	0.719	0.045	0.569
AAI	0.548	0.446	0.636	0.084	0.601
CTDT	0.595	0.496	0.679	0.180	0.616
CTDD	0.593	0.554	0.626	0.182	0.630
AAC	0.584	0.516	0.643	0.160	0.632
PAAC	0.602	0.538	0.657	0.198	0.638
CTD	0.600	0.566	0.629	0.196	0.644
APAAC	0.615	0.533	0.686	0.222	0.645
DPC	0.664	0.616	0.705	0.324	0.697
DDE	0.685	0.602	0.755	0.363	0.726
TPC	0.694	0.670	0.714	0.385	0.758
OPF (This study)	0.745	0.686	0.795	0.487	0.808

**Table 6:** Independent test results of our new feature and conventional feature descriptor.

Feature	ACC	Sn	Sp	MCC	AUC
CTDC	0.562	0.371	0.724	0.101	0.598
PCP	0.552	0.348	0.724	0.078	0.574
AAI	0.598	0.528	0.657	0.187	0.620
CTDT	0.552	0.393	0.686	0.082	0.600
CTDD	0.582	0.472	0.676	0.151	0.618
AAC	0.572	0.528	0.610	0.138	0.593
PAAC	0.577	0.562	0.590	0.152	0.594
CTD	0.582	0.483	0.667	0.152	0.628
APAAC	0.603	0.573	0.629	0.201	0.599
DPC	0.639	0.584	0.686	0.271	0.731
DDE	0.680	0.562	0.781	0.353	0.741
TPC	0.680	0.629	0.724	0.355	0.767
OPF (This study)	0.747	0.742	0.752	0.493	0.827

### 3.5 Characterization of linear T-cell epitopes of hepatitis C virus

**Fig 6:**
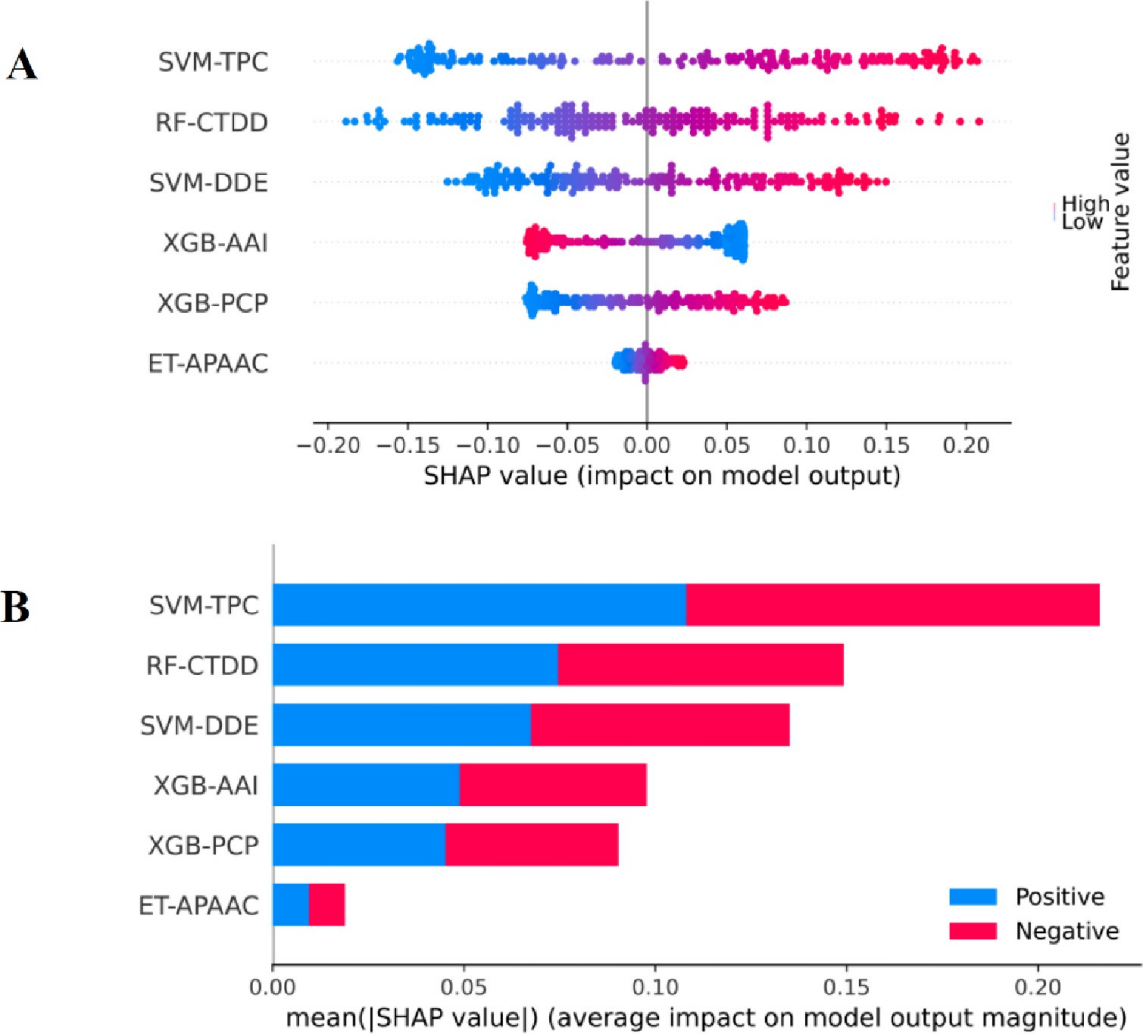
Feature importance analysis for TROLLOPE prediction. (A) Scatter plot of top-15 informative probabilistic features. (B) The average absolute SHAP values of top-15 informative probabilistic features.

**Fig 7:**
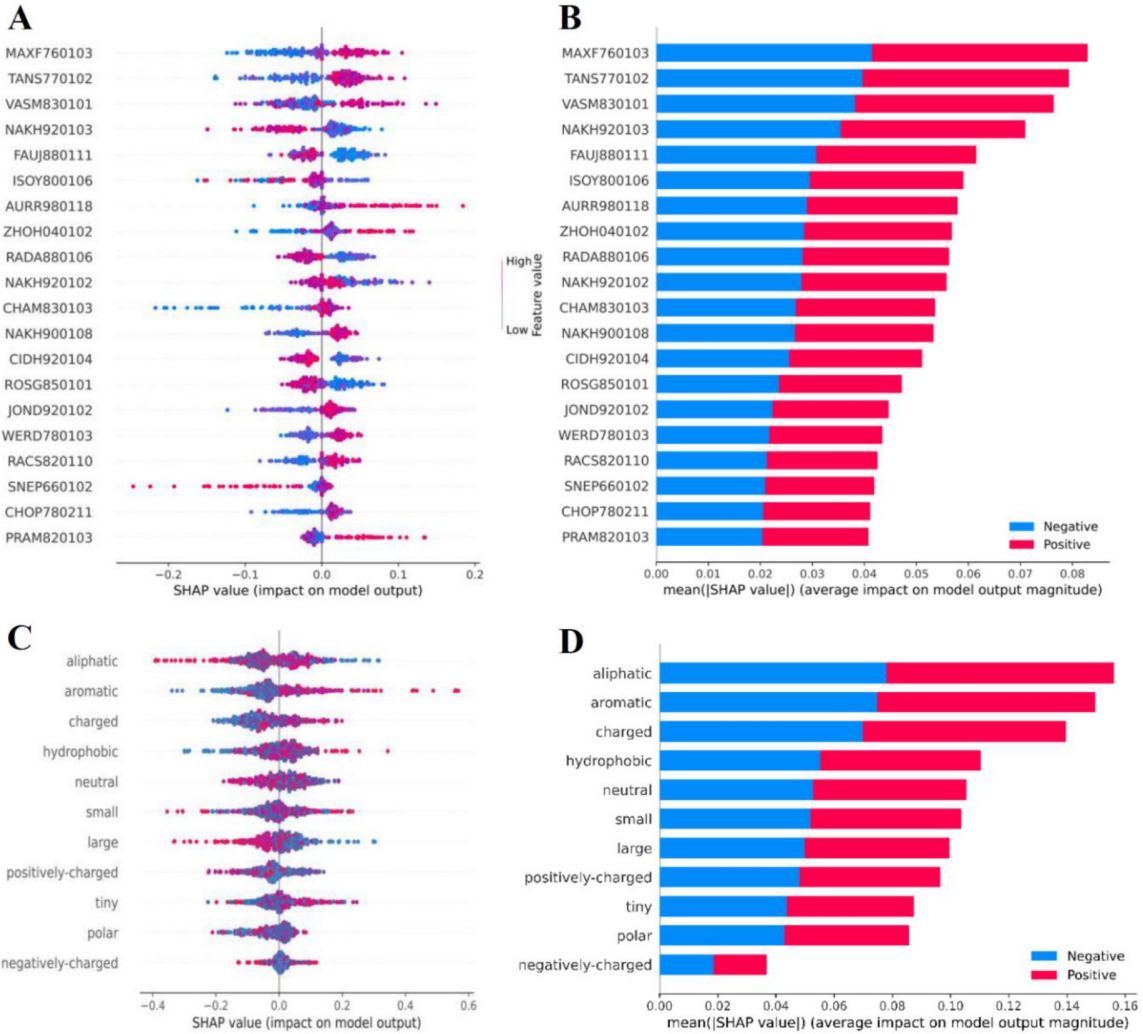
Feature importance analysis for XGB-AAI (A-B) and XGB-PCP (C-D) predictions. (A, C) Scatter plot of top 20 informative features. (B, D) The average absolute SHAP values of top 20 informative features.

The analysis and characterization of feature importance for each type of features are crucial for providing a better understanding of TCE-HCVs. Therefore, we employed an interpretable approach, named the Shapley Additive exPlanations (SHAP), to rank and evaluate the feature importance for TROLLOPE and its constituent base-classifiers. Until now, the SHAP method has been successfully used in various bioinformatics tasks [68–71]. Firstly, the top-six informative probabilistic features of TROLLOPE were assessed for their importance in TCE-HCV identification. **Fig 6** shows the SHAP values of the top-six informative probabilistic features, where positive and negative SHAP values indicate a high probability that the predictions are TCE-HCVs and non-TCE-HCVs, respectively. **Fig 6A** illustrates that most of the top-six informative probabilistic features (with the exception of XGB-AAI) significantly contribute to TCE-HCV prediction, as indicated by their high SHAP values. Secondly, to gain deeper insights into TCE-HCVs, we applied the SHAP method to analyze two of the six base-classifiers (i.e., XGB-AAI and XGB-PCP). Previously, AAI and PCP have been recognized as crucial features for analyzing and charactering various protein functions [72–76]. As seen in **Fig 7**, the important physicochemical properties, such as helical structure conformation (TANS770102, ISOY800106, AURR980118, and WERD780103), beta-sheet structure (CHOP780211) and other conformational characteristics of epitopes (MAXF760103 and VASM830101) play a significant role in TCE-HCV prediction.

Certain amino acid residues in the position preceding a given residue may have specific structural preferences, such as helical propensity or beta-sheet propensity. These important physicochemical properties can provide insights into the preferred amino acids or structural motifs in that position, which can impact the overall conformation and stability of the epitope. Moreover, the hydrophobicity of amino acids within HCV epitopes can significantly influence their structural stability and conformation. In the feature lists of XGB-PCP and XGB-AAI, the presence of "hydrophobic" and "CIDH920104" or Normalized hydrophobicity scales for alpha/beta-proteins (Cid et al., 1992), respectively, indicates that hydrophobic tend to be buried within the protein core, while hydrophilic residues preferentially reside on the protein surface. A balance of hydrophobic and hydrophilic residues within the epitope can contribute to its structural integrity and proper folding, which can potentially influence its antigenicity and immunogenicity [77].

Since aromatic residues have hydrophobic properties, they can participate in hydrophobic interactions with other hydrophobic regions on antibodies or immune receptors. Therefore, the “aromatic” feature from XGB-PCP was also listed in the top ranked feature (**Fig 7C and 7D**). These interactions contribute to the stability and specificity of the antigen-antibody or antigen-receptor binding. In addition, the mean area buried on transfer (ROSG850101) could also be indicative of the hydrophobicity scale of the desired epitopes. Notably, the “aliphatic” feature could also be grouped together with the hydrophobic and aromatic features. The aliphatic residues, such as alanine (Ala), valine (Val), leucine (Leu), and isoleucine (Ile), are non-polar and play important roles in the hydrophobic region of epitopes. These residues have various effects on antigen recognition and immune response [77]. These important features can also influence the exposure and presentation of epitopes on antigens. In some cases, hydrophobic regions within the antigen may be buried within the protein structure, making the epitope less accessible for recognition by immune receptors. Alternatively, hydrophobic patches on the surface of an antigen may be more exposed and accessible, facilitating the binding and recognition of the epitope by immune cells or antibodies.

On the other hand, the charged amino acids, including positively charged (basic) residues such as lysine (Lys) and arginine (Arg), as well as negatively charged (acidic) residues such as aspartic acid (Asp) and glutamic acid (Glu), can also play important roles in epitopes. Interestingly, the “charged”, “polar”, “positively-charged” and “negatively-charged” features from XGB-PCP were also found as the key physicochemical properties in **Fig 7C and 7D**. This evidence was well supported by the FAUJ880111 or Positive charge [78] feature from XGB-AAI prediction (**Fig 7A and 7B**). The presence of charged residues within an epitope can have several effects on antigen recognition and immune response, via electrostatic interactions, to enhance the strength of the binding and play a crucial role in determining the binding affinity and specificity of the epitope [79]. Moreover, charged residues can influence the processing and presentation of epitopes by antigen-presenting cells (APCs) affecting their proteolytic cleavage, degradation, and subsequent presentation on the cell surface via major histocompatibility complex (MHC) molecules [77]. For example, the E2 protein and domain 1 of the HCV-core protein contain frequent positively charged amino acids (Lys and Arg) that are involved in RNA binding, promotes dimerization of the viral RNA, and play a significant role in nucleocapsid (NC) formation and core envelopment by endosomal membranes [80].

In terms of the receptor-epitope binding and specificity on T-cell, NAKH920103 and NAKH920102 represent the role of specific regions on transmembrane proteins (single-spanning proteins). The CYT2 region in NAKH920102 property, which refers to the C-terminal region of a single-spanning protein, may contribute to the amino acid composition within this region, affecting their recognition by antibodies or T-cells [79]. More importantly, the importance of epitope diversity is crucial for vaccine design. The role of potential mutability on HCV epitope function refers to how the mutability, or the propensity to undergo genetic variations, of epitopes can impact their function and interactions with the immune system [81]. The presence of both relative mutability (JOND920102) and relative stability scale extracted from mutation experiments (ZHOH040102) features from XGB-AAI prediction were reasonable. Contributing to this epitope diversity issue, RADA880106, PRAM820103 and SNEP660102 have been reported to capture the variation in amino acid properties that contributes most significantly to the structural diversity of HCV epitopes [39]. Compared to previously reported B-cell epitopes of hepatitis C [39], the feature “Principal component I (PCI) [82]” or SNEP660101 was found to be among the top-10 properties ranked by the accuracy differences. This finding may reflect the different key features that are important for predicting B-cell and T-cell epitopes. On the other hand, the principal component II (PCII) might capture the variation in amino acid properties that contributes most significantly to the structural diversity of HCV epitopes. PCII represents the orthogonal direction to PCI, which generally captures the primary source of variation. PCII can capture additional variations in epitope properties, such as side chain flexibility, polarity, or charge distribution, which can influence the conformational flexibility and structural dynamics of epitopes [83]. Epitopes with different PCII scores may exhibit distinct structural features or conformational preferences, potentially impacting their function and interaction with immune receptors.

### 3.6 Case studies

In this study, we conducted case studies to evaluate the prediction capability of our stacked model TROLLOPE in practical real‐life situations and compared its performance with the top-five base-classifiers. Specifically, we collected six experimentally verified TCE-HCVs from two previous studies [84]. The criteria for HCV epitopes selection in this case study were as follows: 1) they have to be experimentally verified from published research papers, 2) only short peptides (8–11 amino acid residues) that are CD8+ T-cell specific epitopes will be considered (not B-cell specific or CD4+ T-cell epitopes), and 3) these TCE-HCVs should not be found in both the training and independent test datasets. Detailed information about the six TCE-HCVs in the case studies is provided in **S5 Table in S1 File**. In the meanwhile, the prediction results for these TCE-HCVs based on TROLLOPE and the top-five base-classifiers are summarized in **S6 Table in S1 File**. As can be seen, TROLLOPE, along with SVM-TPC and SVM-DDE, can correctly predict all six TCE-HCVs.

Taken together, these findings suggest that TROLLOPE can serve as a useful computational tool for accurately prioritizing high-potential TCE-HCVs from a large number of non-characterized peptides, as evidenced by its performance in both the independent test and case studies.

## 4. Conclusions

This study presents a novel computational approach, termed TROLLOPE, which aims to provide fast and accurate prediction of TCE-HCV. Specifically, we extracted 12 different types of sequence-based feature encoding schemes from several perspectives, such as physicochemical properties, composition-transition-distribution information and composition information, and employed 12 powerful ML algorithms to develop a stacked model. The major contributions of this study are as follows: (i) TROLLOPE is the first computational tool developed specifically for identifying TCE-HCV using sequence information alone; (ii) The new probabilistic features generated based on TROLLOPE offer more discriminative information compared to commonly used feature encodings; (iii) The experimental results, in terms of both cross-validation and independent test results, revealed that TROLLOPE significantly outperformed conventional ML classifiers; and (iv) A user-friendly online web server of TROLLOPE is developed for serving experimental scientists to easily access and utilize the tool for their desired prediction tasks (http://pmlabqsar.pythonanywhere.com/TROLLOPE). It is anticipated that TROLLOPE could be utilized to accelerate the large-scale identification of potential TCE-HCV from non-characterized peptides. However, there are some limitations that can be addressed in future work. Firstly, although our probabilistic features have more discriminative ability in TCE-HCV identification, there is still room for further improvement. For future work, we plan to fuse our probabilistic features with fingerprint descriptors (i.e., Estate, MACCS, and PubChem [85–87]) and sequence-to-vector encodings (i.e., word2vec). Secondly, the performance of TROLLOPE might be improved by combining it with powerful deep learning (DL) approaches, such as deep neural network (DNN) and transfer learning [88, 89]. Thirdly, we are motivated to develop a new ML framework that are capable of identifying multiple viral agents.

## Supporting information

S1 File (DOCX)
