# Supplementary material for: TROLLOPE: A novel sequence-based stacked approach for the accelerated discovery of linear T-cell epitopes of hepatitis C virus
Source: PLoS One. 2023 Aug 25;18(8):e0290538. doi: 10.1371/journal.pone.0290538 (PMC10456195; doi:10.1371/journal.pone.0290538)
Supplement: S1 File — (DOCX) [file pone.0290538.s001.docx]

### **A novel sequence-based stacked approach (TROLLOPE)**

**Input:** Training dataset, $D_{TRN}=\left\{ x_{i}, y_{i} \right\}$

Independent test dataset, $D_{IND}=\left\{ x_{j}, y_{j} \right\}$

**Output:**  Optimal meta-classifier (TROLLOPE)

**The first learning stage**

*for i = 1,…, 144*

Train *i^th^* base-classifier

Evaluate the performance of *i^th^* base-classifier

Generate *i^th^* PF

*End*

Concatenate 144 PFs and convert them into a 140-D probabilistic feature vector

**The second learning stage**

*for i = 1,…, 20*

Generate *i^th^* feature set using the GA-SAR method

Train *i^th^* meta-classifier

Evaluate the performance of *i^th^* meta-classifier

*End*

**Return:**  Optimal meta-classifier (TROLLOPE)


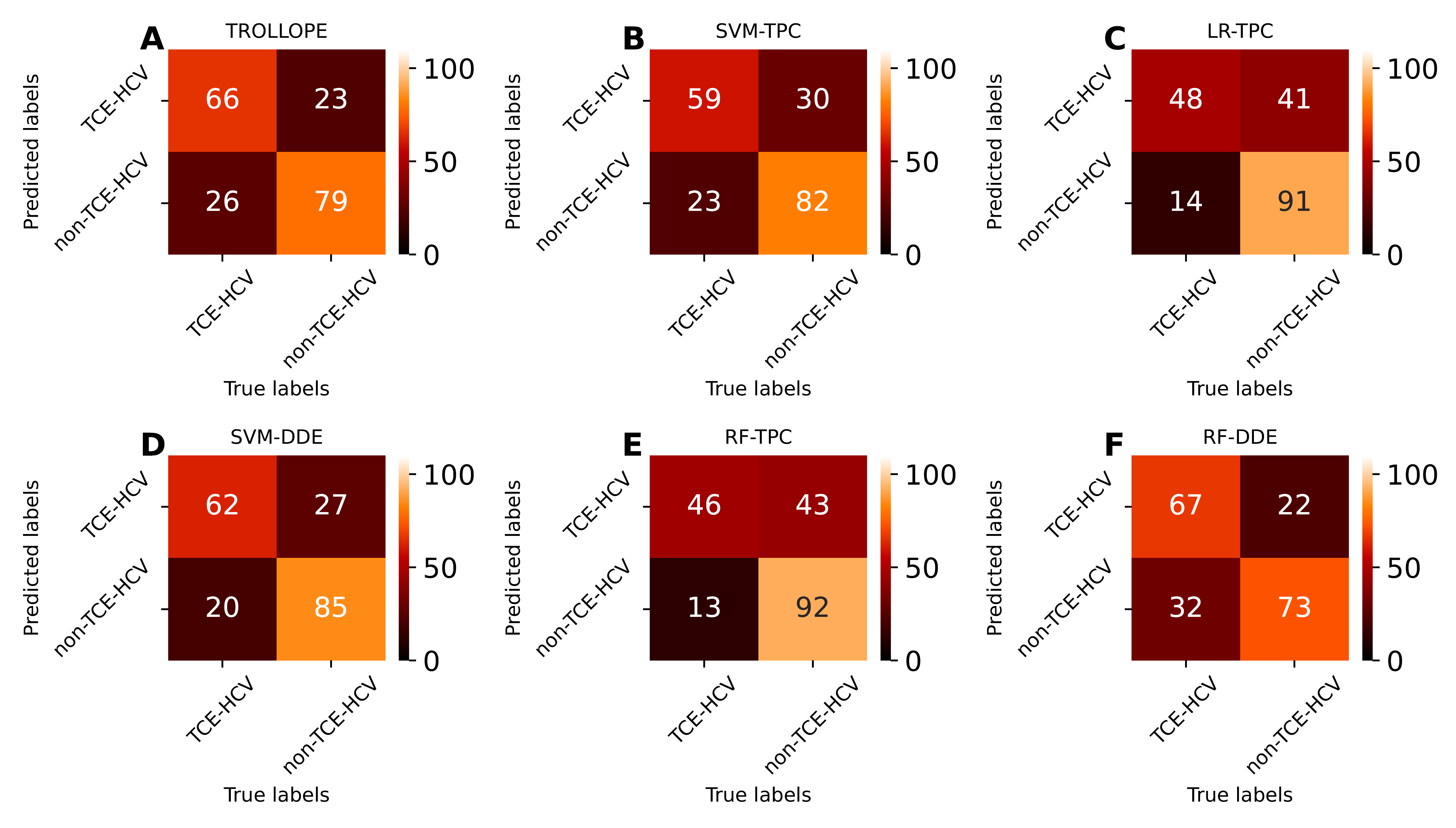


**Figure S1**. Confusion matrices of TROLLOPE **(A)** and top-five base-classifiers **(B-F)** in terms of the independent test dataset.

**Table S1.** Information of parameter settings for twelve ML methods used in this study.

| **Method** | **Parameter** | **Search space** |
| --- | --- | --- |
| ADA | n_estimators | [20, 50, 100, 200, 500] |
| DT | max_depth | 2–20 with an interval of 1. |
| ET | n_estimators | [20, 50, 100, 200, 500] |
| KNN | number of neighbours | 1–150 with an interval of 1 |
| LGBM | n_estimators | [20, 50, 100, 200, 500] |
| LR | C | np.logspace(-3, 3, num=100) |
| MLP | hidden_layer_sizes | [20, 50, 100, 200, 500] |
| NB | var_smoothing | np.logspace(0,-9, num=100) |
| PLS | #Components | 10–1000 with an interval of 10 |
| RF | n_estimators | [20, 50, 100, 200, 500] |
| SVM | Cost | [2^-4^–2^4^] in log_2_ steps |
| XGB | n_estimators | [20, 50, 100, 200, 500] |

Columns 2 and 3 represents the parameter name used in the Scikit-learn library and the range of parameter used to develop the model, respectively.

**Table S2** Cross-validation results of 144 base-classifiers trained with 12 different ML algorithms and 12 feature encoding methods.

| **Method** | **Feature** | **ACC** | **Sn** | **Sp** | **MCC** | **AUC** |
| --- | --- | --- | --- | --- | --- | --- |
| ADA | AAC | 0.656 | 0.610 | 0.695 | 0.309 | 0.708 |
|  | AAI | 0.624 | 0.602 | 0.643 | 0.247 | 0.655 |
|  | APAAC | 0.628 | 0.560 | 0.686 | 0.249 | 0.650 |
|  | CTD | 0.661 | 0.613 | 0.702 | 0.319 | 0.719 |
|  | CTDC | 0.648 | 0.602 | 0.688 | 0.293 | 0.689 |
|  | CTDD | 0.636 | 0.588 | 0.676 | 0.268 | 0.676 |
|  | CTDT | 0.627 | 0.543 | 0.698 | 0.246 | 0.670 |
|  | DDE | 0.658 | 0.695 | 0.626 | 0.321 | 0.684 |
|  | DPC | 0.646 | 0.597 | 0.688 | 0.287 | 0.669 |
|  | PAAC | 0.627 | 0.580 | 0.667 | 0.249 | 0.653 |
|  | PCP | 0.647 | 0.597 | 0.690 | 0.290 | 0.699 |
|  | TPC | 0.668 | 0.530 | 0.786 | 0.328 | 0.719 |
| DT | AAC | 0.647 | 0.669 | 0.629 | 0.299 | 0.675 |
|  | AAI | 0.598 | 0.602 | 0.595 | 0.197 | 0.619 |
|  | APAAC | 0.616 | 0.605 | 0.626 | 0.231 | 0.615 |
|  | CTD | 0.629 | 0.636 | 0.624 | 0.260 | 0.642 |
|  | CTDC | 0.617 | 0.611 | 0.621 | 0.234 | 0.628 |
|  | CTDD | 0.637 | 0.617 | 0.655 | 0.272 | 0.668 |
|  | CTDT | 0.631 | 0.617 | 0.643 | 0.260 | 0.666 |
|  | DDE | 0.658 | 0.633 | 0.679 | 0.313 | 0.656 |
|  | DPC | 0.654 | 0.597 | 0.702 | 0.302 | 0.649 |
|  | PAAC | 0.605 | 0.622 | 0.590 | 0.213 | 0.615 |
|  | PCP | 0.656 | 0.655 | 0.657 | 0.312 | 0.679 |
|  | TPC | 0.674 | 0.577 | 0.757 | 0.341 | 0.667 |
| ET | AAC | 0.671 | 0.633 | 0.702 | 0.338 | 0.757 |
|  | AAI | 0.647 | 0.620 | 0.671 | 0.294 | 0.724 |
|  | APAAC | 0.664 | 0.639 | 0.686 | 0.329 | 0.737 |
|  | CTD | 0.674 | 0.619 | 0.721 | 0.343 | 0.747 |
|  | CTDC | 0.685 | 0.680 | 0.688 | 0.371 | 0.747 |
|  | CTDD | 0.681 | 0.622 | 0.731 | 0.356 | 0.739 |
|  | CTDT | 0.686 | 0.653 | 0.714 | 0.369 | 0.757 |
|  | DDE | 0.672 | 0.664 | 0.679 | 0.345 | 0.749 |
|  | DPC | 0.682 | 0.647 | 0.712 | 0.362 | 0.762 |
|  | PAAC | 0.673 | 0.631 | 0.710 | 0.344 | 0.745 |
|  | PCP | 0.646 | 0.616 | 0.671 | 0.290 | 0.722 |
|  | TPC | 0.698 | 0.591 | 0.788 | 0.391 | 0.753 |
| KNN | AAC | 0.662 | 0.658 | 0.664 | 0.325 | 0.661 |
|  | AAI | 0.651 | 0.661 | 0.643 | 0.305 | 0.652 |
|  | APAAC | 0.665 | 0.655 | 0.674 | 0.332 | 0.665 |
|  | CTD | 0.645 | 0.661 | 0.631 | 0.293 | 0.646 |
|  | CTDC | 0.655 | 0.645 | 0.664 | 0.311 | 0.693 |
|  | CTDD | 0.647 | 0.658 | 0.638 | 0.296 | 0.648 |
|  | CTDT | 0.668 | 0.672 | 0.664 | 0.336 | 0.668 |
|  | DDE | 0.708 | 0.670 | 0.740 | 0.413 | 0.748 |
|  | DPC | 0.703 | 0.633 | 0.762 | 0.401 | 0.757 |
|  | PAAC | 0.676 | 0.678 | 0.674 | 0.353 | 0.725 |
|  | PCP | 0.624 | 0.631 | 0.619 | 0.251 | 0.661 |
|  | TPC | 0.689 | 0.855 | 0.548 | 0.419 | 0.756 |
| LGBM | AAC | 0.663 | 0.613 | 0.705 | 0.322 | 0.729 |
|  | AAI | 0.652 | 0.633 | 0.669 | 0.305 | 0.704 |
|  | APAAC | 0.629 | 0.591 | 0.662 | 0.256 | 0.690 |
|  | CTD | 0.678 | 0.642 | 0.710 | 0.353 | 0.746 |
|  | CTDC | 0.656 | 0.625 | 0.683 | 0.310 | 0.735 |
|  | CTDD | 0.676 | 0.644 | 0.702 | 0.348 | 0.740 |
|  | CTDT | 0.656 | 0.622 | 0.686 | 0.308 | 0.722 |
|  | DDE | 0.683 | 0.630 | 0.729 | 0.362 | 0.738 |
|  | DPC | 0.674 | 0.613 | 0.726 | 0.342 | 0.736 |
|  | PAAC | 0.669 | 0.619 | 0.712 | 0.334 | 0.711 |
|  | PCP | 0.660 | 0.647 | 0.671 | 0.319 | 0.723 |
|  | TPC | 0.569 | 0.073 | 0.990 | 0.166 | 0.534 |
| LR | AAC | 0.586 | 0.502 | 0.657 | 0.161 | 0.635 |
|  | AAI | 0.599 | 0.519 | 0.667 | 0.188 | 0.632 |
|  | APAAC | 0.628 | 0.571 | 0.676 | 0.251 | 0.684 |
|  | CTD | 0.625 | 0.547 | 0.693 | 0.243 | 0.665 |
|  | CTDC | 0.589 | 0.513 | 0.655 | 0.169 | 0.628 |
|  | CTDD | 0.633 | 0.585 | 0.674 | 0.261 | 0.665 |
|  | CTDT | 0.602 | 0.412 | 0.764 | 0.190 | 0.607 |
|  | DDE | 0.682 | 0.605 | 0.748 | 0.357 | 0.732 |
|  | DPC | 0.680 | 0.580 | 0.764 | 0.354 | 0.721 |
|  | PAAC | 0.636 | 0.574 | 0.688 | 0.265 | 0.679 |
|  | PCP | 0.542 | 0.404 | 0.660 | 0.065 | 0.590 |
|  | TPC | 0.705 | 0.552 | 0.836 | 0.409 | 0.786 |
| MLP | AAC | 0.654 | 0.625 | 0.679 | 0.304 | 0.696 |
|  | AAI | 0.638 | 0.625 | 0.650 | 0.282 | 0.699 |
|  | APAAC | 0.651 | 0.605 | 0.690 | 0.299 | 0.693 |
|  | CTD | 0.656 | 0.630 | 0.679 | 0.312 | 0.707 |
|  | CTDC | 0.659 | 0.639 | 0.676 | 0.316 | 0.709 |
|  | CTDD | 0.655 | 0.625 | 0.681 | 0.310 | 0.707 |
|  | CTDT | 0.655 | 0.625 | 0.681 | 0.307 | 0.698 |
|  | DDE | 0.677 | 0.636 | 0.712 | 0.350 | 0.728 |
|  | DPC | 0.660 | 0.622 | 0.693 | 0.319 | 0.704 |
|  | PAAC | 0.659 | 0.633 | 0.681 | 0.315 | 0.698 |
|  | PCP | 0.647 | 0.617 | 0.674 | 0.293 | 0.694 |
|  | TPC | 0.682 | 0.664 | 0.698 | 0.363 | 0.760 |
| NB | AAC | 0.598 | 0.661 | 0.545 | 0.208 | 0.629 |
|  | AAI | 0.558 | 0.776 | 0.374 | 0.162 | 0.602 |
|  | APAAC | 0.613 | 0.655 | 0.576 | 0.233 | 0.652 |
|  | CTD | 0.592 | 0.762 | 0.448 | 0.219 | 0.659 |
|  | CTDC | 0.538 | 0.739 | 0.367 | 0.115 | 0.581 |
|  | CTDD | 0.587 | 0.742 | 0.455 | 0.204 | 0.641 |
|  | CTDT | 0.563 | 0.753 | 0.400 | 0.163 | 0.603 |
|  | DDE | 0.685 | 0.681 | 0.688 | 0.371 | 0.750 |
|  | DPC | 0.676 | 0.718 | 0.640 | 0.360 | 0.727 |
|  | PAAC | 0.605 | 0.650 | 0.567 | 0.217 | 0.636 |
|  | PCP | 0.549 | 0.684 | 0.436 | 0.126 | 0.597 |
|  | TPC | 0.709 | 0.745 | 0.679 | 0.426 | 0.768 |
| PLS | AAC | 0.584 | 0.516 | 0.643 | 0.160 | 0.632 |
|  | AAI | 0.548 | 0.446 | 0.636 | 0.084 | 0.601 |
|  | APAAC | 0.615 | 0.533 | 0.686 | 0.222 | 0.645 |
|  | CTD | 0.600 | 0.566 | 0.629 | 0.196 | 0.644 |
|  | CTDC | 0.538 | 0.401 | 0.655 | 0.056 | 0.558 |
|  | CTDD | 0.593 | 0.554 | 0.626 | 0.182 | 0.630 |
|  | CTDT | 0.595 | 0.496 | 0.679 | 0.180 | 0.616 |
|  | DDE | 0.685 | 0.602 | 0.755 | 0.363 | 0.726 |
|  | DPC | 0.664 | 0.616 | 0.705 | 0.324 | 0.697 |
|  | PAAC | 0.602 | 0.538 | 0.657 | 0.198 | 0.638 |
|  | PCP | 0.538 | 0.325 | 0.719 | 0.045 | 0.569 |
|  | TPC | 0.694 | 0.670 | 0.714 | 0.385 | 0.758 |
| RF | AAC | 0.685 | 0.659 | 0.707 | 0.368 | 0.755 |
|  | AAI | 0.658 | 0.608 | 0.700 | 0.312 | 0.725 |
|  | APAAC | 0.650 | 0.625 | 0.671 | 0.298 | 0.725 |
|  | CTD | 0.690 | 0.692 | 0.688 | 0.381 | 0.743 |
|  | CTDC | 0.668 | 0.630 | 0.700 | 0.333 | 0.748 |
|  | CTDD | 0.695 | 0.658 | 0.726 | 0.386 | 0.759 |
|  | CTDT | 0.686 | 0.681 | 0.690 | 0.372 | 0.746 |
|  | DDE | 0.682 | 0.669 | 0.693 | 0.363 | 0.771 |
|  | DPC | 0.691 | 0.644 | 0.731 | 0.379 | 0.769 |
|  | PAAC | 0.664 | 0.647 | 0.679 | 0.326 | 0.718 |
|  | PCP | 0.660 | 0.636 | 0.681 | 0.320 | 0.731 |
|  | TPC | 0.700 | 0.538 | 0.838 | 0.399 | 0.772 |
| SVM | AAC | 0.664 | 0.639 | 0.686 | 0.327 | 0.710 |
|  | AAI | 0.665 | 0.661 | 0.669 | 0.331 | 0.707 |
|  | APAAC | 0.667 | 0.645 | 0.686 | 0.336 | 0.714 |
|  | CTD | 0.646 | 0.624 | 0.664 | 0.290 | 0.691 |
|  | CTDC | 0.655 | 0.636 | 0.671 | 0.309 | 0.686 |
|  | CTDD | 0.652 | 0.627 | 0.674 | 0.304 | 0.692 |
|  | CTDT | 0.653 | 0.558 | 0.733 | 0.299 | 0.695 |
|  | DDE | 0.699 | 0.639 | 0.750 | 0.394 | 0.780 |
|  | DPC | 0.694 | 0.488 | 0.869 | 0.391 | 0.769 |
|  | PAAC | 0.672 | 0.664 | 0.679 | 0.345 | 0.714 |
|  | PCP | 0.655 | 0.639 | 0.669 | 0.311 | 0.691 |
|  | TPC | 0.696 | 0.440 | 0.914 | 0.407 | 0.791 |
| XGB | AAC | 0.664 | 0.621 | 0.700 | 0.324 | 0.733 |
|  | AAI | 0.627 | 0.577 | 0.669 | 0.248 | 0.683 |
|  | APAAC | 0.627 | 0.574 | 0.671 | 0.248 | 0.684 |
|  | CTD | 0.704 | 0.666 | 0.736 | 0.404 | 0.768 |
|  | CTDC | 0.650 | 0.585 | 0.705 | 0.295 | 0.701 |
|  | CTDD | 0.681 | 0.641 | 0.714 | 0.358 | 0.733 |
|  | CTDT | 0.655 | 0.619 | 0.686 | 0.306 | 0.713 |
|  | DDE | 0.665 | 0.560 | 0.755 | 0.323 | 0.711 |
|  | DPC | 0.656 | 0.521 | 0.771 | 0.306 | 0.701 |
|  | PAAC | 0.650 | 0.597 | 0.695 | 0.295 | 0.689 |
|  | PCP | 0.659 | 0.619 | 0.693 | 0.315 | 0.719 |
|  | TPC | 0.664 | 0.387 | 0.900 | 0.336 | 0.713 |

**Table S3** Independent test results of 144 base-classifiers trained with 12 different ML algorithms and 12 feature encoding methods.

| **Method** | **Feature** | **ACC** | **Sn** | **Sp** | **MCC** | **AUC** |
| --- | --- | --- | --- | --- | --- | --- |
| ADA | AAC | 0.660 | 0.629 | 0.686 | 0.315 | 0.705 |
|  | AAI | 0.629 | 0.573 | 0.676 | 0.250 | 0.672 |
|  | APAAC | 0.593 | 0.551 | 0.629 | 0.179 | 0.646 |
|  | CTD | 0.613 | 0.539 | 0.676 | 0.217 | 0.679 |
|  | CTDC | 0.619 | 0.461 | 0.752 | 0.223 | 0.676 |
|  | CTDD | 0.639 | 0.506 | 0.752 | 0.267 | 0.705 |
|  | CTDT | 0.655 | 0.596 | 0.705 | 0.302 | 0.713 |
|  | DDE | 0.665 | 0.730 | 0.610 | 0.340 | 0.732 |
|  | DPC | 0.675 | 0.640 | 0.705 | 0.346 | 0.750 |
|  | PAAC | 0.660 | 0.629 | 0.686 | 0.315 | 0.682 |
|  | PCP | 0.639 | 0.551 | 0.714 | 0.269 | 0.663 |
|  | TPC | 0.701 | 0.539 | 0.838 | 0.398 | 0.770 |
| DT | AAC | 0.680 | 0.618 | 0.733 | 0.354 | 0.701 |
|  | AAI | 0.572 | 0.607 | 0.543 | 0.149 | 0.618 |
|  | APAAC | 0.593 | 0.517 | 0.657 | 0.176 | 0.587 |
|  | CTD | 0.603 | 0.539 | 0.657 | 0.198 | 0.616 |
|  | CTDC | 0.624 | 0.652 | 0.600 | 0.251 | 0.629 |
|  | CTDD | 0.629 | 0.607 | 0.648 | 0.254 | 0.653 |
|  | CTDT | 0.629 | 0.629 | 0.629 | 0.257 | 0.671 |
|  | DDE | 0.655 | 0.708 | 0.610 | 0.317 | 0.659 |
|  | DPC | 0.665 | 0.652 | 0.676 | 0.327 | 0.664 |
|  | PAAC | 0.557 | 0.551 | 0.562 | 0.112 | 0.575 |
|  | PCP | 0.619 | 0.618 | 0.619 | 0.236 | 0.645 |
|  | TPC | 0.670 | 0.562 | 0.762 | 0.331 | 0.662 |
| ET | AAC | 0.706 | 0.640 | 0.762 | 0.406 | 0.790 |
|  | AAI | 0.680 | 0.663 | 0.695 | 0.358 | 0.757 |
|  | APAAC | 0.706 | 0.640 | 0.762 | 0.406 | 0.771 |
|  | CTD | 0.711 | 0.596 | 0.810 | 0.417 | 0.764 |
|  | CTDC | 0.742 | 0.674 | 0.800 | 0.479 | 0.792 |
|  | CTDD | 0.639 | 0.562 | 0.705 | 0.269 | 0.734 |
|  | CTDT | 0.763 | 0.685 | 0.829 | 0.521 | 0.833 |
|  | DDE | 0.727 | 0.764 | 0.695 | 0.458 | 0.795 |
|  | DPC | 0.737 | 0.685 | 0.781 | 0.469 | 0.816 |
|  | PAAC | 0.696 | 0.618 | 0.762 | 0.385 | 0.776 |
|  | PCP | 0.670 | 0.596 | 0.733 | 0.332 | 0.711 |
|  | TPC | 0.696 | 0.528 | 0.838 | 0.388 | 0.765 |
| KNN | AAC | 0.711 | 0.663 | 0.752 | 0.417 | 0.708 |
|  | AAI | 0.691 | 0.652 | 0.724 | 0.376 | 0.688 |
|  | APAAC | 0.706 | 0.652 | 0.752 | 0.406 | 0.702 |
|  | CTD | 0.655 | 0.618 | 0.686 | 0.304 | 0.652 |
|  | CTDC | 0.639 | 0.618 | 0.657 | 0.275 | 0.695 |
|  | CTDD | 0.675 | 0.640 | 0.705 | 0.346 | 0.673 |
|  | CTDT | 0.732 | 0.708 | 0.752 | 0.460 | 0.730 |
|  | DDE | 0.665 | 0.596 | 0.724 | 0.322 | 0.736 |
|  | DPC | 0.644 | 0.551 | 0.724 | 0.279 | 0.710 |
|  | PAAC | 0.675 | 0.607 | 0.733 | 0.343 | 0.725 |
|  | PCP | 0.644 | 0.596 | 0.686 | 0.282 | 0.678 |
|  | TPC | 0.665 | 0.865 | 0.495 | 0.382 | 0.742 |
| LGBM | AAC | 0.701 | 0.618 | 0.771 | 0.395 | 0.756 |
|  | AAI | 0.680 | 0.663 | 0.695 | 0.358 | 0.738 |
|  | APAAC | 0.665 | 0.584 | 0.733 | 0.321 | 0.721 |
|  | CTD | 0.660 | 0.573 | 0.733 | 0.311 | 0.754 |
|  | CTDC | 0.655 | 0.607 | 0.695 | 0.303 | 0.728 |
|  | CTDD | 0.680 | 0.573 | 0.771 | 0.352 | 0.751 |
|  | CTDT | 0.747 | 0.742 | 0.752 | 0.493 | 0.812 |
|  | DDE | 0.711 | 0.674 | 0.743 | 0.418 | 0.764 |
|  | DPC | 0.644 | 0.551 | 0.724 | 0.279 | 0.739 |
|  | PAAC | 0.644 | 0.551 | 0.724 | 0.279 | 0.723 |
|  | PCP | 0.634 | 0.562 | 0.695 | 0.259 | 0.686 |
|  | TPC | 0.572 | 0.079 | 0.990 | 0.173 | 0.530 |
| LR | AAC | 0.582 | 0.528 | 0.629 | 0.157 | 0.599 |
|  | AAI | 0.582 | 0.551 | 0.610 | 0.160 | 0.603 |
|  | APAAC | 0.613 | 0.483 | 0.724 | 0.213 | 0.650 |
|  | CTD | 0.598 | 0.506 | 0.676 | 0.184 | 0.638 |
|  | CTDC | 0.572 | 0.539 | 0.600 | 0.139 | 0.595 |
|  | CTDD | 0.593 | 0.506 | 0.667 | 0.174 | 0.650 |
|  | CTDT | 0.552 | 0.326 | 0.743 | 0.076 | 0.599 |
|  | DDE | 0.686 | 0.551 | 0.800 | 0.364 | 0.753 |
|  | DPC | 0.660 | 0.562 | 0.743 | 0.310 | 0.754 |
|  | PAAC | 0.577 | 0.461 | 0.676 | 0.140 | 0.629 |
|  | PCP | 0.546 | 0.393 | 0.676 | 0.072 | 0.564 |
|  | TPC | 0.716 | 0.539 | 0.867 | 0.434 | 0.796 |
| MLP | AAC | 0.691 | 0.663 | 0.714 | 0.377 | 0.717 |
|  | AAI | 0.608 | 0.742 | 0.495 | 0.242 | 0.665 |
|  | APAAC | 0.619 | 0.539 | 0.686 | 0.227 | 0.679 |
|  | CTD | 0.629 | 0.562 | 0.686 | 0.249 | 0.678 |
|  | CTDC | 0.655 | 0.663 | 0.648 | 0.309 | 0.709 |
|  | CTDD | 0.598 | 0.551 | 0.638 | 0.189 | 0.666 |
|  | CTDT | 0.675 | 0.674 | 0.676 | 0.349 | 0.745 |
|  | DDE | 0.701 | 0.629 | 0.762 | 0.395 | 0.786 |
|  | DPC | 0.727 | 0.652 | 0.790 | 0.448 | 0.776 |
|  | PAAC | 0.670 | 0.618 | 0.714 | 0.334 | 0.719 |
|  | PCP | 0.613 | 0.517 | 0.695 | 0.216 | 0.673 |
|  | TPC | 0.670 | 0.618 | 0.714 | 0.334 | 0.757 |
| NB | AAC | 0.644 | 0.730 | 0.571 | 0.303 | 0.642 |
|  | AAI | 0.582 | 0.775 | 0.419 | 0.206 | 0.638 |
|  | APAAC | 0.608 | 0.652 | 0.571 | 0.223 | 0.645 |
|  | CTD | 0.546 | 0.685 | 0.429 | 0.117 | 0.636 |
|  | CTDC | 0.552 | 0.764 | 0.371 | 0.146 | 0.599 |
|  | CTDD | 0.552 | 0.663 | 0.457 | 0.122 | 0.617 |
|  | CTDT | 0.521 | 0.674 | 0.390 | 0.067 | 0.582 |
|  | DDE | 0.706 | 0.640 | 0.762 | 0.406 | 0.793 |
|  | DPC | 0.680 | 0.742 | 0.629 | 0.370 | 0.755 |
|  | PAAC | 0.613 | 0.674 | 0.562 | 0.236 | 0.637 |
|  | PCP | 0.541 | 0.663 | 0.438 | 0.103 | 0.597 |
|  | TPC | 0.670 | 0.708 | 0.638 | 0.345 | 0.749 |
| PLS | AAC | 0.572 | 0.528 | 0.610 | 0.138 | 0.593 |
|  | AAI | 0.598 | 0.528 | 0.657 | 0.187 | 0.620 |
|  | APAAC | 0.603 | 0.573 | 0.629 | 0.201 | 0.599 |
|  | CTD | 0.582 | 0.483 | 0.667 | 0.152 | 0.628 |
|  | CTDC | 0.562 | 0.371 | 0.724 | 0.101 | 0.598 |
|  | CTDD | 0.582 | 0.472 | 0.676 | 0.151 | 0.618 |
|  | CTDT | 0.552 | 0.393 | 0.686 | 0.082 | 0.600 |
|  | DDE | 0.680 | 0.562 | 0.781 | 0.353 | 0.741 |
|  | DPC | 0.639 | 0.584 | 0.686 | 0.271 | 0.731 |
|  | PAAC | 0.577 | 0.562 | 0.590 | 0.152 | 0.594 |
|  | PCP | 0.552 | 0.348 | 0.724 | 0.078 | 0.574 |
|  | TPC | 0.680 | 0.629 | 0.724 | 0.355 | 0.767 |
| RF | AAC | 0.706 | 0.618 | 0.781 | 0.405 | 0.810 |
|  | AAI | 0.670 | 0.640 | 0.695 | 0.336 | 0.734 |
|  | APAAC | 0.634 | 0.573 | 0.686 | 0.260 | 0.709 |
|  | CTD | 0.711 | 0.685 | 0.733 | 0.419 | 0.771 |
|  | CTDC | 0.696 | 0.629 | 0.752 | 0.385 | 0.782 |
|  | CTDD | 0.660 | 0.607 | 0.705 | 0.313 | 0.756 |
|  | CTDT | 0.701 | 0.652 | 0.743 | 0.396 | 0.783 |
|  | DDE | 0.722 | 0.753 | 0.695 | 0.447 | 0.799 |
|  | DPC | 0.727 | 0.674 | 0.771 | 0.448 | 0.816 |
|  | PAAC | 0.660 | 0.629 | 0.686 | 0.315 | 0.739 |
|  | PCP | 0.655 | 0.596 | 0.705 | 0.302 | 0.712 |
|  | TPC | 0.711 | 0.517 | 0.876 | 0.426 | 0.786 |
| SVM | AAC | 0.655 | 0.618 | 0.686 | 0.304 | 0.700 |
|  | AAI | 0.660 | 0.629 | 0.686 | 0.315 | 0.674 |
|  | APAAC | 0.670 | 0.562 | 0.762 | 0.331 | 0.721 |
|  | CTD | 0.629 | 0.562 | 0.686 | 0.249 | 0.672 |
|  | CTDC | 0.655 | 0.652 | 0.657 | 0.308 | 0.688 |
|  | CTDD | 0.619 | 0.573 | 0.657 | 0.231 | 0.666 |
|  | CTDT | 0.670 | 0.528 | 0.790 | 0.332 | 0.744 |
|  | DDE | 0.758 | 0.697 | 0.810 | 0.511 | 0.822 |
|  | DPC | 0.686 | 0.427 | 0.905 | 0.383 | 0.777 |
|  | PAAC | 0.706 | 0.663 | 0.743 | 0.407 | 0.729 |
|  | PCP | 0.629 | 0.596 | 0.657 | 0.253 | 0.675 |
|  | TPC | 0.727 | 0.663 | 0.781 | 0.448 | 0.798 |
| XGB | AAC | 0.680 | 0.596 | 0.752 | 0.353 | 0.762 |
|  | AAI | 0.696 | 0.674 | 0.714 | 0.388 | 0.728 |
|  | APAAC | 0.649 | 0.618 | 0.676 | 0.294 | 0.698 |
|  | CTD | 0.660 | 0.584 | 0.724 | 0.311 | 0.730 |
|  | CTDC | 0.603 | 0.506 | 0.686 | 0.194 | 0.694 |
|  | CTDD | 0.665 | 0.584 | 0.733 | 0.321 | 0.723 |
|  | CTDT | 0.716 | 0.685 | 0.743 | 0.429 | 0.770 |
|  | DDE | 0.680 | 0.562 | 0.781 | 0.353 | 0.754 |
|  | DPC | 0.655 | 0.494 | 0.790 | 0.300 | 0.741 |
|  | PAAC | 0.624 | 0.573 | 0.667 | 0.240 | 0.697 |
|  | PCP | 0.686 | 0.607 | 0.752 | 0.364 | 0.727 |
|  | TPC | 0.675 | 0.393 | 0.914 | 0.366 | 0.760 |

**Table S4** Performance Evaluation of BLAST-based predictors using different *E*-values.

| **E-Value** | **ACC** | **Sn** | **Sp** | **MCC** |
| --- | --- | --- | --- | --- |
| 0.1 | 0.644 | 0.663 | 0.629 | 0.291 |
| 0.01 | 0.675 | 0.506 | 0.819 | 0.344 |
| 0.001 | 0.577 | 0.090 | 0.990 | 0.190 |
| 0.0001 | 0.541 | 0.000 | 1.000 | 0.000 |

**Table S5.** Detailed information of all peptides in the case studies.

| **#** | **Peptide ID** | **Sequence** | **Reference** |
| --- | --- | --- | --- |
| 1 | 408-428_H77c | KQNIQLINTNGSWHINSTALN | [1] |
| 2 | 408-428_S52 | KQKLQLVNTNGSWHINSTALN | [1] |
| 3 | 430-451_H77c | NESLNTGWLAGLFYQHKFNSSG | [1] |
| 4 | 430-451_S52 | NESINTGFIAGLFYYHKFNSTG | [1] |
| 5 | 523-549_H77c | GAPTYSWGANDTDVFVLNNTRPPLGNW | [1] |
| 6 | 523-549_S52 | GRPTYNWGENETDVFLLESLRPPSGRW | [1] |

**Table S6.** Detailed prediction results of PSATTCA and top five ML classifiers on the case studies

| **#** | **True** | TROLLOPE | SVM-TPC | LR-TPC | SVM-DDE | RF-TPC | RF-DDE |
| --- | --- | --- | --- | --- | --- | --- | --- |
| 1 | TCE-HCV | TCE-HCV | TCE-HCV | non-TCE-HCV | TCE-HCV | non-TCE-HCV | non-TCE-HCV |
| 2 | TCE-HCV | TCE-HCV | TCE-HCV | non-TCE-HCV | TCE-HCV | non-TCE-HCV | TCE-HCV |
| 3 | TCE-HCV | TCE-HCV | TCE-HCV | non-TCE-HCV | TCE-HCV | non-TCE-HCV | non-TCE-HCV |
| 4 | TCE-HCV | TCE-HCV | TCE-HCV | non-TCE-HCV | TCE-HCV | non-TCE-HCV | non-TCE-HCV |
| 5 | TCE-HCV | TCE-HCV | TCE-HCV | TCE-HCV | TCE-HCV | non-TCE-HCV | TCE-HCV |
| 6 | TCE-HCV | TCE-HCV | TCE-HCV | non-TCE-HCV | TCE-HCV | non-TCE-HCV | non-TCE-HCV |

## References

[1] T. Donnison *et al.*, "A pan‐genotype hepatitis C virus viral vector vaccine generates T cells and neutralizing antibodies in mice," *Hepatology,* vol. 76, no. 4, pp. 1190-1202, 2022.
